# The Clinical Utility of Lower Extremity Dual-Energy CT Angiography in the Detection of Bone Marrow Edema in Diabetic Patients with Peripheral Artery Disease

**DOI:** 10.3390/jcm13061536

**Published:** 2024-03-07

**Authors:** Chiara Floridi, Laura Maria Cacioppa, Tommaso Valeri, Nicolo Rossini, Marzia Rosati, Vincenzo Vento, Alessandro Felicioli, Marco Macchini, Roberto Candelari, Marina Carotti, Andrea Giovagnoni

**Affiliations:** 1 Department of Clinical, Special and Dental Sciences, University Politecnica delle Marche, 60126 Ancona, Italy; c.floridi@staff.univpm.it (C.F.); l.m.cacioppa@univpm.it (L.M.C.); s1103602@pm.univpm.it (T.V.); m.carotti@staff.univpm.it (M.C.); a.giovagnoni@staff.univpm.it (A.G.); 2 Division of Interventional Radiology, Department of Radiological Sciences, University Hospital “Azienda Ospedaliera Universitaria delle Marche”, 60126 Ancona, Italy; marzia.rosati@ospedaliriuniti.marche.it (M.R.); alessandro.felicioli@ospedaliriuniti.marche.it (A.F.); marco.macchini@ospedaliriuniti.marche.it (M.M.); r.candelari@ospedaliriuniti.marche.it (R.C.); 3 Division of Radiology, Department of Radiological Sciences, University Hospital “Azienda Ospedaliera Universitaria delle Marche”, 60126 Ancona, Italy; 4 Vascular Surgery Unit, Aortic Team, Lancisi Cardiovascular Center, 60122 Ancona, Italy; vincenzovento1987@gmail.com

**Keywords:** computed tomography angiography, peripheral arterial disease, dual-energy computed tomography, digital subtraction angiography, bone marrow edema, diabetes

## Abstract

(1) **Background**: Type 2 diabetes is a major cause of incidences and the progression of peripheral artery disease (PAD). Bone marrow edema (BME) is an important finding suggestive of underlying bone inflammation in non-traumatic diabetic patients with PAD. Our aim was to evaluate the presence, severity, and clinical implications of BME detected by virtual non-calcium application (VNCa) of dual-energy CT angiography (DE-CTA). (2) **Methods**: A consecutive series of 76 diabetic patients (55 men; mean age 71.6 ± 11.2 yrs) submitted to lower limb DE-CTA for PAD evaluation and revascularization planning, which were retrospectively analyzed. VNCa images were independently and blindly revised for the presence, location, and severity of BME by two radiologists with 10 years of experience. BME and non-BME groups were evaluated in terms of PAD clinical severity and 6-month secondary major amputation rate. (3) **Results**: BME was present in 17 (22%) cases, while 59 (78%) patients were non-BME. The BME group showed a significantly higher incidence of major amputation (*p* < 0.001) and a significantly higher number of patients with advanced clinical stages of PAD compared to the non-BME group (*p* = 0.024). (4) **Conclusions**: Lower limb DE-CTA with VNCa application is a useful tool in the detection of BME in diabetic patients with PAD, simultaneously enabling the evaluation of the severity and location of the arterial disease for revascularization planning. BME presence could be a marker of clinically severe PAD and a possible risk factor for revascularization failure.

## 1. Introduction

Chronic arterial occlusive disease of the lower extremities, also known as peripheral arterial disease (PAD), is a spectrum of diseases characterized by atheromatous stenosis or occlusion of arterial vessel tracts from pedal arteries up to aortoiliac axis [1,2]. PAD is one of the prevalent causes of hospitalization and mortality and represents the third most important cause of atherosclerotic morbidity after coronary heart and cerebrovascular diseases [3,4]. Several certain and modifiable risk factors for PAD, such as male sex, family history, black ethnicity, hyperlipidemia, smoking, and primary hypertension, have been widely recognized [5,6,7]. Type 2 diabetes (DM 2) is greatly involved in PAD pathophysiology, leading to an increase in PAD incidence, as well as in disease progression [6,7]. Furthermore, the duration and severity of hyperglycemia have been found to be related to the extent of vascular disease [5] and to the presence of diabetic foot ulcers, which are at risk of increasing throughout one’s life and should be appropriately classified by the recently developed WIfI (Wound, Ischemia, and Foot Infection) scoring system. This classification includes the three most important parameters involved in lower limb amputation risk and covers diabetic patients, who were previously excluded from the concept of critical limb ischemia due to the more complex clinical condition. WifI classification provides an earlier risk stratification of threatened lower limbs and helps in clinical and therapeutic decision-making, overcoming the angiosome concept [8,9]. It is well known that chronic hyperglycemia causes arterial blood vessel damage, worsening claudication, and ischemic rest pain in PAD patients [10]. A timely and focused correction of glucose hematic levels is necessary to avoid gangrene and ulcers [10]. When developed, both PAD and diabetes conditions could be cause of amputation.

PAD has also been described as an independent factor for amputation in patients with diabetes. At the same time, the microvascular dysfunction related to type II diabetes acts as a synergistic factor of PAD in promoting lower limb amputation [11]. For this reason, microvascular disease prevention and management have acquired a central role in PAD patient’s treatment.

The diagnostic workup for PAD includes, after physical examination, duplex ultrasound (DUS) as a first step for PAD screening and detection, followed by a second step of diagnostic modality with a panoramic view such as computed tomography angiography (CTA), magnetic resonance angiography (MRA), or digital subtraction angiography (DSA) for the evaluation of arterial stenosis and occlusion in terms of site, extension, and severity [1,5,12].

Currently, DSA maintains its role mainly for therapeutic interventional purposes or in the case of equivocal imaging results, while non-invasive imaging exams are currently preferred for purely diagnostic purposes [4,5].

In this regard, CTA of the lower limbs has gained a major role as a diagnostic tool for PAD due to its safety, great availability, high spatial and contrast resolution, rapid acquisition protocols, and the possibility to evaluate the whole arterial tree of a certain anatomic district [4,13]. Regarding diagnostic accuracy, CTA has been demonstrated to be highly accurate (overall sensitivity 95–97%, specificity 91–98%) in the assessment of hemodynamically significant stenosis and occlusion, especially for larger proximal arteries [13,14,15]. CTA potential is lower for small distal limbs and widely calcified arteries; however, its diagnostic accuracy below the knee remains acceptable [4,14,16].

Another advantage of CTA is the capability to evaluate either the luminal lesion, the arterial wall, or the surrounding soft tissues with comparable accuracy [16,17,18].

Currently, the nearly isotropic voxel available in modern scanners and several reconstruction techniques such as multiplanar reconstruction (MPR), maximal intensity projection (MIP), volume rendering (VR), and curved planar reformation (CPR) enables an angiographic-like visualization of the arterial lumen. Those techniques are extremely effective for procedural guidance and planning [19].

A new era of CT imaging is dual-energy computed tomography (DECT), whose use and availability have increased in recent years in many clinical applications. In contrast to single-spectrum imaging, DECT exploits atomic number and chemical composition to distinguish materials with similar attenuation coefficients. Iodine, calcium, and other materials with high atomic numbers can be easily differentiated. This mechanism allows different useful applications of DECT in PAD, also reducing iodine contrast medium dose and radiation burden [20,21,22,23]. Furthermore, DECT post-processing algorithms enable the removal of bone and the calcium content of highly calcified plaques, reducing “blooming” artifacts and obtaining a true CTA “luminogram” [18,24,25,26].

The virtual non-calcium technique (VNCa) is another outstanding application of DE imaging currently used in musculoskeletal radiology.

The VNCa image is produced by means of a three-material decomposition model, which enables bone mineral and gross calcifications removal, thus displaying the underlying bone marrow without superposition [27,28,29,30]. With VNCa imaging, the different attenuation values of the bone marrow reflect its water and fat content. Both qualitative analyses, by means of color-coded maps, and quantitative evaluations, by means of regions of interest (ROI), may be performed on VNCa images with the aim of detecting bone marrow edema (BME) [27,31,32].

Several causes of BME have been described in the literature, including vitamin D deficiency, traumas, osteoporosis, chronic liver failure, DM 2, and PAD [33,34].

Compared to MRI, VNCa showed similar results in detecting BME, either in traumatic or non-traumatic settings [35,36,37]. BME is commonly developed by diabetic patients with PAD, most likely due to both infectious or dysmetabolic non-medullary osteitis and infectious involvement of the medullary cavity, also known as osteomyelitis [38,39,40].

All these aspects suggest that DE-CTA performed in diabetic patients with PAD could be used at the same time to evaluate PAD severity as well as the presence of lower limb BME by employing the VNCa algorithm during a single examination. The detection of positive or negative BME may have a consequent impact on PAD management.

The purpose of our study was to assess the presence and severity of BME in diabetic patients submitted to a single lower limb DE-CTA exam for PAD evaluation. As secondary endpoints, we evaluated the possible correlations of BME with PAD clinical severity and secondary limb loss rate.

## 2. Materials and Methods

### 2.1. Patient Population and Study Design

This retrospective single-center study was approved by the Internal Review Board (IRB).

A DE-CTA examination informed consent was obtained from all the participants.

All the consecutive patients submitted to DE-CTA between September 2021 and May 2023 were collected and reviewed. All patients were previously referred to the Vascular Surgery or Vascular Medicine Departments of our hospital and submitted to a vascular evaluation for PAD consisting of patient anamnesis, physical evaluation with ankle–brachial index (ABI) measurement, DUS examination, and laboratory testing including blood glucose, glycohemoglobin, and renal function. To be further included in the analysis, patients had a >10-year history of DM 2 and were diagnosed with symptomatic PAD from severe claudication and rest pain to non-healing ulcers with necrosis or gangrene (Fontaine categories IIB, III, and IV, respectively).

All the selected cases were submitted to endovascular or surgical revascularization within one month of the DE-CTA examination.

Bone and wound cultures, plain radiographs, ongoing or previous broad-spectrum intravenous antibiotic therapy, and previous minor amputations performed on the examined side were investigated. All patients with documented osteomyelitis history due to infected ulcers were excluded.

Known rheumatological disease, diseases affecting calcium and phosphorus metabolism, major lower limb traumas in the previous six months, severe osteoporosis, documented allergies to iodine or to iodine-derived medium of contrast, renal impairment with values <45 mL/min of glomerular filtration rate, and congestive heart failure were also considered as exclusion criteria. Other excluded cases were those with clear clinical symptoms of acute limb ischemia [41]. Furthermore, all patients who lacked imaging follow-up or were missing physical examinations performed in our institution were excluded.

All patients were followed up by clinical examination at 1,3, 6, and 12 months after being submitted to any revascularization procedure.

Secondary major amputation was defined, according to ESC guidelines, as a failed attempt at revascularization with no possibility of reintervention and limb deterioration due to infection or necrosis despite optimal management [19] recorded within 6 months from intervention.

### 2.2. CT Study Protocol

A third-generation dual-source DECT scanner (Siemens Somatom Force, Siemens Healthcare, Forchheim, Germany) was used for CT examinations. Supine patient positioning, with feet first, was used in all cases. An adhesive tape was used to approach, block, and stabilize feet during the exam in order to reduce movement artifacts. Iodine contrast medium was always administered with an intravenous injection using an 18-gauge cannula placed in a superficial vein of the antecubital fossa or forearm. Craniocaudal scanning direction was used for DE-CTA image acquisition. Scan length was extended from the infrarenal abdominal aorta up to the toes. All the exams were acquired with a standardized DE-CTA acquisition protocol, consisting of a 120 KVp bolus-tracking acquisition (CARE Bolus, Siemens, Munich, Germany) to initiate scanning 8 s after reaching the trigger threshold of 300 Hounsfield unit (HU) in a region of interest (ROI) placed in the infrarenal abdominal aorta. The two tubes, A and B, worked with 90 and 150 kVp, respectively. Image quality reference was set to 120-67 mAs. DE-CTA acquisition and scanning parameter reports are presented in Table 1.

An iodine delivery rate of 1.4–1.8 was reached in all included cases by intravenously administering 1 mL/kg of iodinated contrast medium volume followed by 40 mL of saline solution, both at a flow rate ≥ 4 mL/s.

Automatic exposure control (CareDose 4DTM, Siemens Healthcare, Forchheim, Germany) was used in each acquisition protocol to adapt the tube current to variation in patient attenuation, either between different patients or within any patient.

### 2.3. DE-CTA Image Reconstruction and Post-Processing

Low and high kVp DE-CTA data (90 kVp dataset and Sn150 kVp dataset, respectively) coupled with a weighted average or mixed dataset from both tubes were reconstructed using a medium sharp convolution kernel (Qr 40). The weighted average was used to simulate and replace a conventional CT scan.

The DE-CTA scans were post-processed on a dedicated advanced workstation (Syngo MMWP version VA 20; Siemens Healthcare, Forchheim, Germany). VNCa images were generated using a three-material decomposition algorithm. A specific bone marrow edema application, capable of differentiating yellow bone marrow, red bone marrow, and bone minerals, was applied. Calcium was also subtracted from CT images. Thresholds of 100 HU to a maximum of 800 HU and default settings for color-coded maps of bone marrow between −150 HU and 100 HU were applied. VNCa images were then displayed as color-coded maps and overlayed both on 2D weighted average CT images and on 3D imaging.

Multiplanar reformats (MPR; thickness, 1.5 mm; increment, 1.0 mm) and maximum intensity projections (MIP; thickness, 10.0 mm; increment, 1.0 mm) in transverse, oblique coronal, and sagittal planes were obtained.

**Figure 1:**
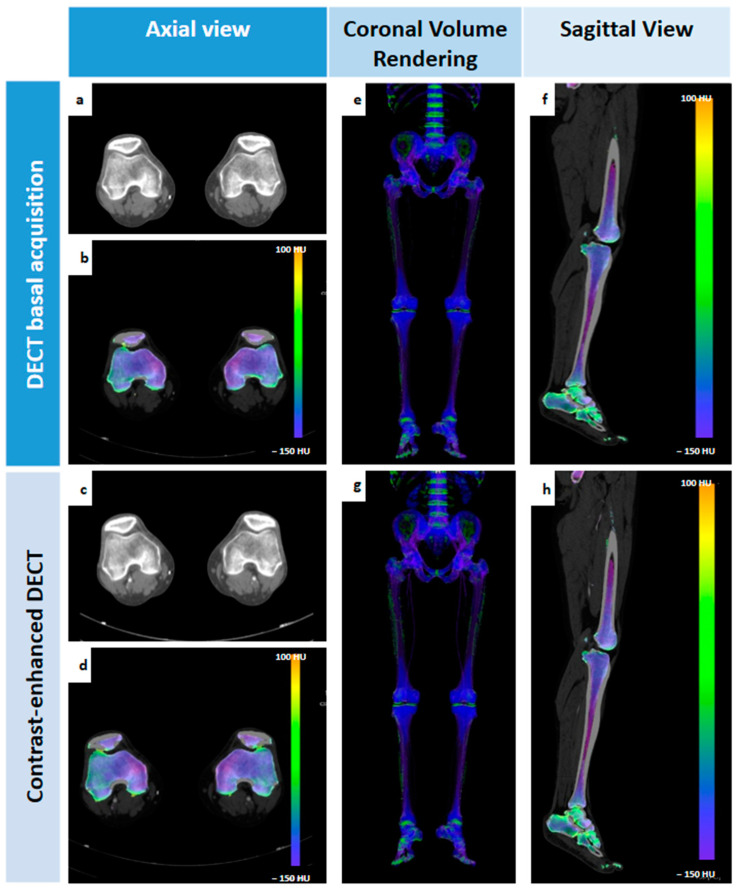
DECT axial unenhanced (**a**), mixed 120 KvP image of the lower limbs at knee level with its corresponding VNCa image (**b**), and analogous DE-CTA axial mixed 120 KvP image (**c**) with its corresponding VNCa image (**d**), showing no qualitative differences in the colorimetric map of bone marrow attenuation. Again, no visual differences are present between unenhanced (**e**) and enhanced (**g**) 3D volume rendering images and between unenhanced (**f**) and enhanced (**h**) sagittal reformatted colorimetric maps of bone marrow attenuation.

In the first ten cases, the VNCa algorithm was preliminarily applied to baseline non-contrast DECT acquisitions, showing qualitative and quantitative results comparable to those obtained in DE-CTA images (Figure 1).

### 2.4. Image Analysis

Images were independently assessed for the presence, location, and severity of BME by two expert radiologists in vascular and musculoskeletal radiology, both with at least 10 years of experience in these fields. Both the investigators were blind to sex, age, patient history, and radiology reports. The images were anonymized and displayed in random order to reduce reader bias. In cases of conflicting results, the positive finding of BME underwent a second analysis. For color-coded 2D images, the presence of BME was displayed as green-yellow colored (attenuation values around 0 HU) or as yellow-red colored for higher attenuation values. Lower attenuation values, negative for BME, were displayed as blue-purple colored. A qualitative assessment of BME severity was also performed by means of a color scale (green, yellow, and red were evaluated as mild, moderate, and severe BME, respectively) on the 2D colorimetric maps displayed in the three planes. On 3D images, a blue-green scale was also displayed, showing edema as green and normal bone marrow as blue. In addition, the DE-CTA mixed images were examined for the presence and location of arterial stenoses.

### 2.5. Statistical Analysis

GraphPad Prism version 9.1.1 (GraphPad Software, Boston, MA, USA) statistical software was used for data analysis. Numerical variables were reported as average value and range. The difference between BME and non-BME groups was tested for significance using a paired-sample *t*-test in terms of the presence and severity of BME, location and severity of PAD, and the performed revascularization strategy (interventional radiology endovascular treatment vs. amputation vs. surgical revascularization). Accordingly, a paired measures test (*t*-test) was also used to compare the location of BME and clinical severity of PAD (Fontaine classification). A *p* < 0.05 was considered statistically significant. Inter-observer agreement was measured using Cohen’s k test and interpreted as follows: for values between 0.01 and 0.20, the agreement was poor; for values between 0.21 and 0.40, the agreement was modest; for values between 0.41 and 0.6, the agreement was moderate; for values between 0.61 and 0.80, the agreement was substantial; and for values between 0.8 and 1, the agreement was excellent.

## 3. Results

Out of the total 101 patients who fulfilled the inclusion criteria of our study, we analyzed a final population of 76 patients, as the remaining 25 were excluded due to the exclusion criteria listed above. Out of the total 76 patients, 55 were males (median age 74 yrs, range 45–85 yrs), and 21 were females (median age 74 yrs, range 41–92 yrs). The calculated median body mass index (BMI) was 25.7 (range 17–33.5). The symptoms of presentation of PAD were classified according to Fontaine grading. A total of 30 patients (39.5%) suffered from severe claudication, 18 (23.7%) patients had ischemic rest pain, and 28 (36.8%) presented tissue loss (ulceration or gangrene).

Demographic and Fontaine grading of the patients included in this study were reported in Table 2.

No adverse events occurred during DE-CTA examinations in all the included cases. No significant motion artifacts were registered, and the VNC algorithm was successfully applied in all cases.

The interobserver agreement was excellent for the evaluation of BME presence (Cohen’s k = 0.83) and modest (Cohen’s k = 0.27) for BME severity.

Out of a total of 76 DE-CTA VNCa color-coded maps analyzed, 17 (22%) cases were considered positive for BME, and 59 (78%) were considered non-BME.

**Figure 2:**
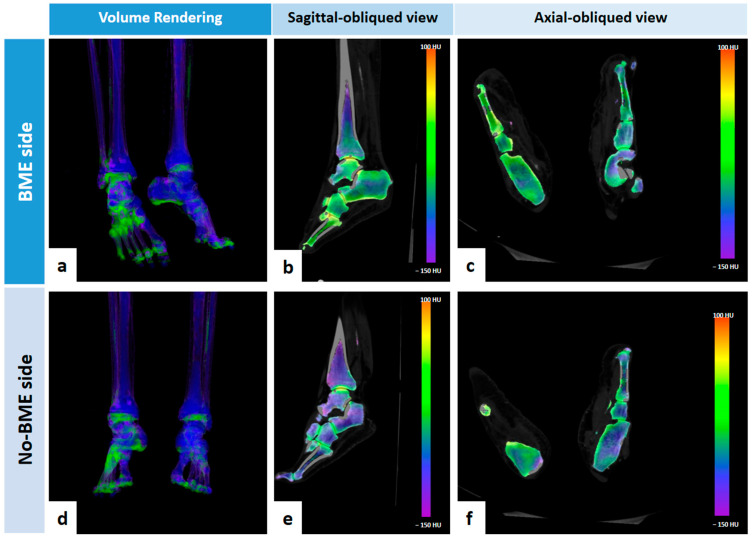
Seventy-six-year-old male patients with stage 4 PAD and BME in right midfoot (fourth and fifth metatarsal bones), as shown in 3D volume rendering image (**a**), in reformatted sagittal oblique VNCa 2D image (**b**) and reformatted axial oblique VNCa 2D image (**c**). No signs of BME are present on contralateral side images (**d**–**f**).

In nine (52.9%) cases, BME was located in the phalanges: four (23.5%) in the proximal, two (11.8%) in the middle, and three (17.6%) in the distal phalanges. In two (11.8%) cases, BME was located in metatarsal bones (Figure 2), and in five (29.4%) cases, BME was located in tarsal bones (all cases in multiple bone segments except for one case located in calcaneal bone). In one case, an area of BME was found in the distal part of the tibial bone.

Regarding the qualitative analysis of BME severity, in fifteen (88%) cases, the BME was evaluated as mild, one case (5.9%) was evaluated as moderate, and one case (5.9%) was evaluated as severe.

Within one month after the DE-CTA scan, 50 (65.8%) patients were submitted to endovascular revascularization consisting in all cases of percutaneous transluminal angioplasty (PTA), while in 26 (34.2%) cases, surgical revascularization was preferred.

As shown in Table 3, no significant differences in terms of demographic factors between the BME and non-BME groups were recorded. No significant differences between the BME group and non-BME group were also observed in terms of endovascular vs. surgical revascularization (*p* = 0.908).

**Figure 3:**
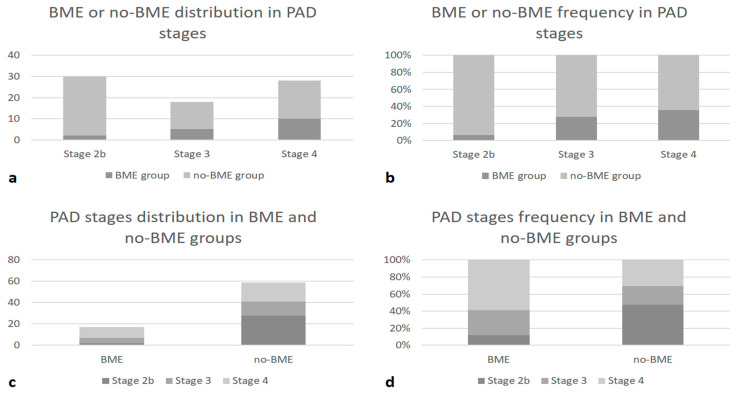
Bar graphs showing (**a**) BME and non-BME numeric distribution in different PAD clinical stages; (**b**) BME and non-BME percentage frequency in crescent PAD clinical stages; (**c**) numeric distribution of PAD stages among the group of BME and non-BME patients; (**d**) percentage frequency of PAD stages in BME and non-BME groups.

Regarding the severity of PAD, in the positive-BME group, a significantly higher number of patients was classified as Fontaine clinical stage III and IV compared to the non-BME group (*p* = 0.024). These results are summarized in Figure 3.

A statistically significant difference between the BME and non-BME groups was instead found in terms of the incidence of a 6-month limb loss rate on the examined side. In particular, the VNCa positive-BME group showed a significantly higher incidence of major amputation compared to the non-BME group (*p* < 0.001).

**Figure 4:**
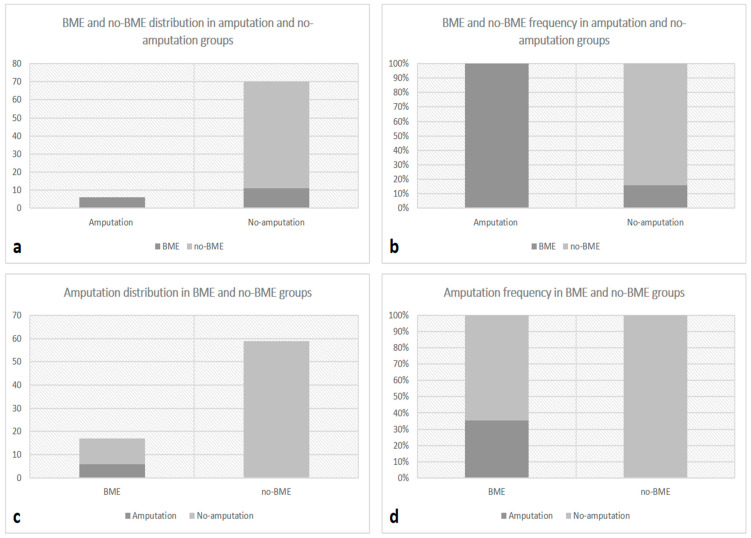
Bar graphs showing BME and non-BME numeric distribution in patients with and without secondary major amputation at 6-month follow-up (**a**) and the relative percentage frequency (**b**) and distribution of secondary major amputation at 6-month follow-up between BME and non-BME groups (**c**) and its relative frequency (**d**).

These results are illustrated in Figure 4.

When considering the qualitative assessment of BME severity within the BME group, no statistically significant differences were found in terms of clinical severity of PAD and BME location (*p* = 0.948 and *p* = 0.06, respectively).

## 4. Discussion

CTA has been widely recommended as the first-line examination for the evaluation of the extent and severity of PAD, effectively replacing DSA as treatment planning guidance [19,42]. While the current literature mainly focused on the advantages of DE-CTA in the evaluation of arterial lumen, in the estimation of arterial stenosis and in-stent patency evaluation, its ability to recognize lower limbs BME in diabetic patients with PAD has been poorly investigated [12,13,24,25,39,43]. The abnormal fluid signal within the bone marrow, routinely detected on MRI, is a nonspecific, yet important, finding suggestive of underlying bone inflammation in non-traumatic diabetic patients [33,36,40,43,44]. Third-generation DECT and its advanced material decomposition algorithms may be an alternative tool in assessing BME due to their good diagnostic performance compared to MRI [32,33,34,35,36].

**Figure 5:**
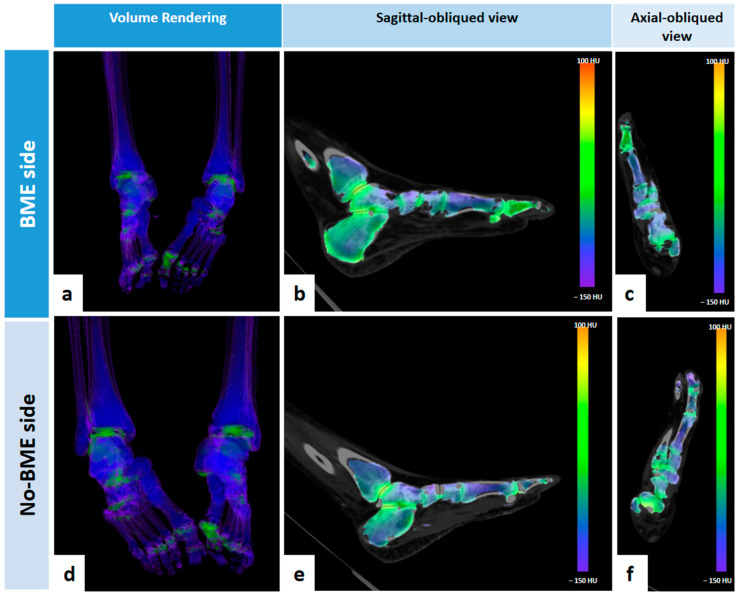
Seventy-six-year-old female patient with stage III PAD and BME in the first proximal phalange of the left foot, as shown in 3D volume rendering image (**a**), in reformatted sagittal oblique VNCa 2D image (**b**) and reformatted axial oblique VNCa 2D image (**c**). No signs of BME are present on the contralateral foot in the same anatomical location (**d**–**f**).

This retrospective study of 76 patients aimed to assess the clinical value of BME detected by VNCa reconstructions in symptomatic diabetic patients submitted to lower limb DE-CTA for PAD definition (Figure 5).

In previous studies, BME evaluation was mainly performed by non-contrast DECT [33,34,35,36,37,38], while none, as far as we know, focused on the application of the VNCa algorithm on DE-CTA images. VNCa-DECTA enables us to simultaneously evaluate the severity and extent of PAD and the presence of BME as a complication of both arterial and metabolic diseases, providing important information for medical and surgical treatment management of this difficult category of patients.

The suggested DE-CTA acquisition and post-processing protocol also provide clinical practices the undeniable advantage of performing a single-shot exam to evaluate two different clinical conditions, reducing workload and healthcare costs and avoiding MRI drawbacks such as multiple contraindications, long waiting times, and discomfort for patients.

A previous study with interesting results documented the correlation between edematous bone marrow changes in the bones adjacent to diabetic ulcers and suspected osteomyelitis, confirming the remarkable value of VNCa-DECT in the quantitative assessment of BME in diabetic foot ulcers [39]. In these series, DE-CTA imaging was applied to detect BME of suspected infectious origin due to the presence of water influx and granulation tissue, and, therefore, highly increasing CT attenuation values. VNCa-DECT showed high diagnostic accuracy for osteomyelitis and has proven to be an important aid for medical and surgical decisions consisting of intravenous antibiotics and minor amputations [39]. It is also known how both arterial and diabetic diseases, leading to local ischemic inflammation, ulcers, infection, and repeated microtraumas, have been proven to be directly and indirectly responsible for BME pathogenesis [33,45].

In our series, the role of VNCa-DECT was focused on the correlation between the detection of BME and the clinical severity of PAD. As expected, the presence of BME was observed in a significantly higher number of patients with advanced clinical stages of PAD (Fontaine III and IV), confirming how diabetes and PAD are both involved in BME development, independently from the presence of osteomyelitis. In the present study, patients with clinical and radiological findings of overt osteomyelitis had been preliminarily excluded, and BME was observed in bone structures not necessarily adjacent to cutaneous diabetic ulcers. Furthermore, BME was, in most cases (88%), a mild entity, suggesting a multifactorial inflammation rather than a pure infective origin.

Taking into consideration that BME and non-BME groups were homogeneous in terms of selected revascularization strategy, the presence of BME was significantly associated with a higher 6-month limb loss rate.

A higher amputation risk has been largely observed in patients with diabetic foot syndrome, especially with longer duration, due to the adverse prognostic impact of several comorbidities and damage to multiple organs, such as chronic kidney disease. Furthermore, the delayed diagnostic workup due to the absence of typical symptoms and the distally localized and highly calcified arterial lesions often make revascularization attempts unsatisfactory [7,46,47,48].

Despite the previously demonstrated incremental impact of DM on short- and long-term limb salvaging in patients with PAD [47], BME may be considered an adjunctive, easily detectable risk factor for future limb loss and useful information given to clinicians for better multidisciplinary case management. A closer follow-up of VNCa-positive patients and earlier therapeutic strategies of revascularization could help in avoiding subsequent major amputations and significant systemic complications.

Several important limitations must be considered. Firstly, the single-center and retrospective nature of this study is subject to selection bias, a limited number of patients, and the use of clinical criteria (Fontaine classification) as reference standards. Secondly, a qualitative evaluation rather than a quantitative analysis of DE-CTA images for the detection of BME by the VNCa algorithm was performed. The qualitative evaluation was preferred with the aim of avoiding the drawback of the use of different cut-offs. The lack of an additional quantitative analysis is certainly a major limitation. Nevertheless, as also reported by Suh et al. [49], the qualitative analysis demonstrated a superior diagnostic performance compared to the quantitative assessment of BME by means of VNCa. The lack of a correlation with MRI findings is certainly another limitation. Due to the retrospective design of this study, MR imaging for too many patients resulted in a lack of correlation with DE-CT sensitivity and specificity for the detection of BME. Furthermore, the statistical analysis was only focused on clinical outcome PAD-related endpoints, and these results are only applicable to the vendor-specific DE-CTA technique and VNCa post-processing software (Syngo MMWP version VA 20; Siemens Healthcare, Forchheim, Germany).

Further analyses focused on the correlation between occluded arterial segments and the location of BME would help to emphasize the role of PAD as a risk factor for the development of BME. As a future perspective, this analysis will also be completed with specific information about the type and location of diabetic ulcers.

In conclusion, the present series is one of the few in the literature to apply VNC color-coded maps for the detection of BME to DE-CTA performed for PAD treatment planning. These initial results appear encouraging and useful in optimizing the difficult therapeutic management of diabetic patients with PAD, identifying BME as an early risk factor for future limb loss.

The value of DE-CTA to evaluate both vascular pathology and BME at the same time, thus reducing time to diagnose and provide treatment, costs, and patient discomfort, should be confirmed in larger and prospective studies.

## Figures and Tables

**Table 1:** Dual-energy CTA scanning parameters.

DE-CTA Acquisition Protocol	
KVp	Tube A 90 kVp Tube B 150 kVp
mAs	120-67
Collimation	1 mm (acquisition 192 × 2 × 0.6 mm^2^)
Rotation time	0.5 s
Pitch	0.4
FOV	300 mm
Reconstruction thickness	1 mm
Contrast medium volume	1 mL/Kg
Contrast medium infusion rate	≥4 mL/s
Iodine delivery rate (IDR)	1.4–1.8

**Table 2:** Demographics and clinical characteristics of the study population.

Demographic	
Patients (n)	76
Age (yrs)	74 (41–92)
Weight (kg)	77 (53–126)
Height (cm)	171 (158–193)
BMI (kg/m^2^)	25.7 (17–33.5)
**Clinical (PAD Fontaine stage)**	**n° (%)**
Stage IIB (claudication at a distance < 200 m)	30 (39.5%)
Stage III (rest pain)	18 (23.7%)
Stage IV (ulcers, necrosis, and/or gangrene)	28 (36.8%)

**Table 3:** Comparison of patients’ general and clinical characteristics between BME and non-BME groups. ns: not significant.

Parameter	BME-Group (n = 17)	Non-BME-Group (n = 59)	*p*
Patients (n)	17 (22%)	59 (78%)	
Age (yrs)	71.5 ± 10.4	71.9 ± 11.0	ns
Male/female (ratio)	12/5	43/16	ns
BMI (kg/m^2^)	25.5	25.7	ns
Fontaine stage III/IV (%)	15	31	0.024
Endovascular revascularization (%)	11	39	ns
Surgical revascularization (%)	6	20	ns
Secondary amputation (%)	6	0	<0.001

## Data Availability

Data are contained within the article.

## References

[1] Yadav V., Khanduri S., Yadav P., Pandey S., Tyagi E., Yadav H., Krishnam A., Hamza M. (2020). Diagnostic Accuracy of Color Doppler and Calcium Scoring versus Dual-Energy Computed Tomography Angiography in the Assessment of Peripheral Arterial Diseases of Lower Limb. J. Clin. Imaging Sci..

[2] Aday A.W., Matsushita K. (2021). Epidemiology of Peripheral Artery Disease and Polyvascular Disease. Circ. Res..

[3] Criqui M.H., Aboyans V. (2015). Epidemiology of peripheral artery disease. Circ. Res..

[4] Shwaiki O., Rashwan B., Fink M.A., Kirksey L., Gadani S., Karuppasamy K., Melzig C., Thompson D., D’Amico G., Rengier F. (2021). Lower extremity CT angiography in peripheral arterial disease: From the established approach to evolving technical developments. Int. J. Cardiovasc. Imaging.

[5] Conte M.S., Bradbury A.W., Kolh P., White J.V., Dick F., Fitridge R., Mills J.L., Ricco J.-B., Suresh K.R., Murad M.H. (2019). Global Vascular Guidelines on the Management of Chronic Limb-Threatening Ischemia. Eur. J. Vasc. Endovasc. Surg..

[6] Beckman J.A., Creager M.A., Libby P. (2002). Diabetes and atherosclerosis: Epidemiology, pathophysiology, and management. JAMA.

[7] Barnes J.A., Eid M.A., Creager M.A., Goodney P.P. (2020). Epidemiology and Risk of Amputation in Patients with Diabetes Mellitus and Peripheral Artery Disease. Arter. Thromb. Vasc. Biol..

[8] Schaper N.C., van Netten J.J., Apelqvist J., Bus S.A., Hinchliffe R.J., Lipsky B.A., IWGDF Editorial Board (2020). Practical Guidelines on the prevention and management of diabetic foot disease (IWGDF 2019 update). Diabetes Metab. Res. Rev..

[9] Monteiro-Soares M., Russell D., Boyko E.J., Jeffcoate W., Mills J.L., Morbach S., Game F., on behalf of the International Working Group on the Diabetic Foot (IWGDF) (2020). Guidelines on the classification of diabetic foot ulcers (IWGDF 2019). Diabetes/Metabolism Res. Rev..

[10] Tao G.L., Zheng L.S., Wang Z.Y., Xu Y.P., Zhong X.G., Wang H., Zhang X.S. (2023). Clinical characteristics and risk factors of diabetic foot ulcers with PAD. Eur. Rev. Med. Pharmacol. Sci..

[11] Behroozian A., Beckman J.A. (2020). Microvascular Disease Increases Amputation in Patients with Peripheral Artery Disease. Arter. Thromb. Vasc. Biol..

[12] Floridi C., Cacioppa L.M., Agliata G., Cellina M., Rossini N., Valeri T., Curzi M., Felicioli A., Bruno A., Rosati M. (2023). True Non-Contrast Phase versus Virtual-Non Contrast: “Lights and Shadows” of Dual Energy CT Angiography in Peripheral Arterial Disease. Appl. Sci..

[13] Napoli A., Anzidei M., Zaccagna F., Marincola B.C., Zini C., Brachetti G., Cartocci G., Fanelli F., Catalano C., Passariello R. (2011). Peripheral arterial occlusive disease: Diagnostic performance and effect on therapeutic management of 64-section CT angiography. Radiology.

[14] Itoga N.K., Kim T., Sailer A.M., Fleischmann D., Mell M.W. (2017). Lower extremity computed tomography angiography can help predict technical success of endovascular revascularization in the superficial femoral and popliteal artery. J. Vasc. Surg..

[15] Horehledova B., Mihl C., Milanese G., Brans R., Eijsvoogel N.G., Hendriks B.M.F., Wildberger J.E., Das M. (2018). CT Angiography in the Lower Extremity Peripheral Artery Disease Feasibility of an Ultra-Low Volume Contrast Media Protocol. Cardiovasc. Interv. Radiol..

[16] Met R., Bipat S., Legemate D.A., Reekers J.A., Koelemay M.J.W. (2009). Diagnostic performance of computed tomography angiography in peripheral arterial disease: A systematic review and meta-analysis. JAMA.

[17] Takx R.A., Partovi S., Ghoshhajra B.B. (2016). Imaging of atherosclerosis. Int. J. Cardiovasc. Imaging.

[18] Meyersohn N.M., Walker T.G., Oliveira G.R. (2015). Advances in axial imaging of peripheral vascular disease. Curr. Cardiol. Rep..

[19] Aboyans V., Ricco J.B., Bartelink M.E.L., Björck M., Brodmann M., Cohnert T., Collet J.P., Czerny M., De Carlo M., Debus S. (2018). 2017 ESC Guidelines on the Diagnosis and Treatment of Peripheral Arterial Diseases, in collaboration with the European Society for Vascular Surgery (ESVS): Document covering atherosclerotic disease of extracranial carotid and vertebral, mesenteric, renal, upper and lower extremity arteries Endorsed by: The European Stroke Organization (ESO) the Task Force for the Diagnosis and Treatment of Peripheral Arterial Diseases of the European Society of Cardiology (ESC) and of the European Society for Vascular Surgery (ESVS). Eur. Heart J..

[20] Tagliati C., Lanza C., Pieroni G., Amici L., Carotti M., Giuseppetti G.M., Giovagnoni A. (2021). Ultra-low-dose chest CT in adult patients with cystic fibrosis using a third-generation dual-source CT scanner. La Radiol. Med..

[21] Agostini A., Borgheresi A., Mari A., Floridi C., Bruno F., Carotti M., Schicchi N., Barile A., Maggi S., Giovagnoni A. (2019). Dual-energy CT: Theoretical principles and clinical applications. La Radiol. Med..

[22] Sanghavi P.S., Jankharia B.G. (2019). Applications of dual energy CT in clinical practice: A pictorial essay. Indian J. Radiol. Imaging.

[23] Odedra D., Narayanasamy S., Sabongui S., Priya S., Krishna S., Sheikh A. (2022). Dual Energy CT Physics—A Primer for the Emergency Radiologist. Front. Radiol..

[24] Koo B.J., Won J.H., Choi H.C., Na J.B., Kim J.E., Park M.J., Jo S.H., Park H.O., Lee C.E., Kim M.J. (2022). Automatic Plaque Removal Using Dual-Energy Computed Tomography Angiography: Diagnostic Accuracy and Utility in Patients with Peripheral Artery Disease. Medicina.

[25] Kim J.S., Park S.H., Park S., Hwang J.H., Kim J.H., Pak S.Y., Lee K., Schmidt B. (2022). Imaging Findings of Peripheral Arterial Disease on Lower-Extremity CT Angiography Using a Virtual Monoenergetic Imaging Algorithm. J. Korean Soc. Radiol..

[26] Kosmala A., Weng A.M., Schmid A., Gruschwitz P., Grunz J., Bley T.A., Petritsch B. (2022). Dual-Energy CT Angiography in Peripheral Arterial Occlusive Disease: Diagnostic Accuracy of Different Image Reconstruction Approaches. Acad. Radiol..

[27] Carotti M., Salaffi F., Beci G., Giovagnoni A. (2019). The application of dual-energy computed tomography in the diagnosis of musculoskeletal disorders: A review of current concepts and applications. La Radiol. Med..

[28] Foti G., Serra G., Iacono V., Marocco S., Bertoli G., Gori S., Zorzi C. (2021). Identification of Non-Traumatic Bone Marrow Oedema: The Pearls and Pitfalls of Dual-Energy CT (DECT). Tomography.

[29] Ren Q., Tang D., Xiong Z., Zhao H., Zhang S. (2022). Traumatic bone marrow lesions in dual-energy computed tomography. Insights Imaging.

[30] Foti G., Lombardo F., Guerriero M., Rodella T., Cicciò C., Faccioli N., Serra G., Manenti G. (2022). Management of vertebral compression fractures: The role of dual-energy CT in clinical practice. La Radiol. Med..

[31] Cellina M., Cè M., Rossini N., Cacioppa L.M., Ascenti V., Carrafiello G., Floridi C. (2023). Computed Tomography Urography: State of the Art and Beyond. Tomography.

[32] Foti G., Mantovani W., Faccioli N., Crivellari G., Romano L., Zorzi C., Carbognin G. (2021). Identification of bone marrow edema of the knee: Diagnostic accuracy of dual-energy CT in comparison with MRI. La Radiol. Med..

[33] Foti G., Guerriero M., Faccioli N., Fighera A., Romano L., Zorzi C., Carbognin G. (2021). Identification of bone marrow edema around the ankle joint in non-traumatic patients: Diagnostic accuracy of dual-energy computed tomography. Clin. Imaging.

[34] Jans L., De Kock I., Herregods N., Verstraete K.L., Van den Bosch F.E., Carron P., Oei E.H., Elewaut D., Jacques P. (2018). Du-al-energy CT: A new imaging modality for bone marrow oedema in rheumatoid arthritis. Ann. Rheum. Dis..

[35] Wu H., Zhang G., Shi L., Li X., Chen M., Huang X., Cao X., Tan S., Cui Y., Liang C. (2019). Axial Spondyloarthritis: Dual-Energy Virtual Noncalcium CT in the Detection of Bone Marrow Edema in the Sacroiliac Joints. Radiology.

[36] Saba L., De Filippo M., Saba F., Fellini F., Marcy P.-Y., Dagan R., Voituriez P., Aelvoet J., Klotz G., Bernard R. (2019). Dual energy CT and research of the bone marrow edema: Comparison with MRI imaging. Indian J. Radiol. Imaging.

[37] Li M., Qu Y., Song B. (2017). Meta-analysis of dual-energy computed tomography virtual non-calcium imaging to detect bone marrow edema. Eur. J. Radiol..

[38] Foti G., Longo C., Sorgato C., Oliboni E.S., Mazzi C., Motta L., Bertoli G., Marocco S. (2023). Osteomyelitis of the Lower Limb: Diagnostic Accuracy of Dual-Energy CT versus MRI. Diagnostics.

[39] Mens M.A., de Geus A., Wellenberg R.H.H., Streekstra G.J., Weil N.L., Bus S.A., Busch-Westbroek T.E., Nieuwdorp M., Maas M. (2023). Preliminary evaluation of dual-energy CT to quantitatively assess bone marrow edema in patients with diabetic foot ulcers and suspected osteomyelitis. Eur. Radiol..

[40] Donovan A., Schweitzer M.E. (2010). Use of MR imaging in diagnosing diabetes-related pedal osteomyelitis. RadioGraphics.

[41] Björck M., Earnshaw J.J., Acosta S., Gonçalves F.B., Cochennec F., Debus E.S., Hinchliffe R., Jongkind V., Koelemay M.J.W., Menyhei G. (2020). Editor’s Choice—European Society for Vascular Surgery (ESVS) 2020 Clinical Practice Guidelines on the Management of Acute Limb Ischaemia. Eur. J. Vasc. Endovasc. Surg..

[42] Gerhard-Herman M.D., Gornik H.L., Barrett C., Barshes N.R., Corriere M.A., Drachman D.E., Fleisher L.A., Fowkes F.G., Hamburg N.M., Kinlay S. (2017). 2016 AHA/ACC Guideline on the Management of Patients with Lower Extremity Peripheral Artery Disease: Executive Summary: A Report of the American College of Cardiology/American Heart Association Task Force on Clinical Practice Guidelines. Circulation.

[43] De Santis D., De Cecco C.N., Schoepf U.J., Nance J.W., Yamada R.T., Thomas B.A., Otani K., Jacobs B.E., Turner D.A., Wichmann J.L. (2019). Modified calcium subtraction in dual-energy CT angiography of the lower extremity runoff: Impact on di-agnostic accuracy for stenosis detection. Eur. Radiol..

[44] Roug I.K., Pierre-Jerome C. (2012). MRI spectrum of bone changes in the diabetic foot. Eur. J. Radiol..

[45] Gonzalez-Martín D., Herrera-Perez M., Martín-Velez P., Rendon-Díaz D. (2019). Prevalence of bone marrow edema in a study population with foot and/or ankle pain. Foot.

[46] Richter L., Freisinger E., Lüders F., Gebauer K., Meyborg M., Malyar N.M. (2018). Impact of diabetes type on treatment and outcome of patients with peripheral artery disease. Diabetes Vasc. Dis. Res..

[47] Vacirca A., Faggioli G., Pini R., Gallitto E., Mascoli C., Cacioppa L.M., Gargiulo M., Stella A. (2019). The Outcome of Technical Intraoperative Complications Occurring in Standard Aortic Endovascular Repair. Ann. Vasc. Surg..

[48] Lowry D., Saeed M., Narendran P., Tiwari A. (2018). A review of distribution of atherosclerosis in the lower limb arteries of patients with diabetes mellitus and peripheral vascular disease. Vasc. Endovasc. Surg..

[49] Suh C.H., Yun S.J., Jin W., Lee S.H., Park S.Y., Ryu C.W. (2018). Diagnostic performance of dual-energy CT for the detection of bone marrow oedema: A systematic review and meta-analysis. Eur. Radiol..

